# Facets of Parenting a Child with Hypoplastic Left Heart Syndrome

**DOI:** 10.1155/2012/714178

**Published:** 2012-04-02

**Authors:** Gwen R. Rempel, Laura G. Rogers, Vinitha Ravindran, Joyce Magill-Evans

**Affiliations:** ^1^Faculty of Nursing, University of Alberta, Edmonton, AB, Canada; ^2^Department of Pediatrics, Faculty of Medicine and Dentistry, University of Alberta, Edmonton, AB, Canada; ^3^Faculty of Rehabilitation Medicine, University of Alberta, Edmonton, AB, Canada

## Abstract

The purpose of the study was to conceptualize the needs of parents of young children with hypoplastic left heart syndrome (HLHS) to provide a theoretical framework to inform the development of future parent interventions. Participants were parents and grandparents (*n* = 53) of 15 young children who had undergone the Sano surgical approach for HLHS. Analysis of recorded and transcribed single interviews with each participant was done as directed by interpretive description methodology. A model of five facets of parenting was conceptualized. These included survival parenting, “hands-off” parenting, expert parenting, uncertain parenting, and supported parenting. The facets of parenting delineated through this study provide a theoretical framework that can be used to guide the development and evaluation of interventions for parents of children with complex congenital heart disease and potentially other life-threatening conditions. Each facet constitutes a critical component for educational or psychosocial intervention for parents.

## 1. Introduction

The challenges parents face when a child has a chronic health condition are extensively described in the literature. The development and implementation of evidence-informed practices and approaches to improve outcomes for chronically ill children and their families are facilitated by a programmatic approach to research. A review of nursing research that addressed the fragility of the parent-child relationship related to the child or parent's chronic condition revealed that many of the studies represented only a single investigation [1]. Multiple studies within a program that lead to theory development such as “special needs parenting” [2] and “compensatory parenting” [3] or to a typology of family management [4, 5] and subsequent tool development (e.g., family management measure [6, 7]) are becoming more common in child health research. Translating this type of evidence into child and family nursing practice through intervention research is slowly emerging, with more progress in some speciality areas than others (e.g., childhood cancer [8, 9]).

Although congenital heart disease (CHD) is the most commonly occurring congenital malformation [10, 11] and hypoplastic left heart syndrome (HLHS), without treatment, would be responsible for 25–40% of all neonatal cardiac deaths [12], evidence regarding interventions to support parents is lacking. Descriptive studies of the responses of parents to their child's CHD and ongoing treatment within a context of uncertainty are numerous. Many studies provide evidence of pathology highlighting parental fear [13–16], parenting stress [17–21], maternal anxiety and depressed mood [22], and parental distress and hopelessness [23, 24]. Theoretical models, however, to inform intervention are lacking.

Concerning parenting children with HLHS, the most severe and life-threatening form of CHD, several studies within a program of research entitled “Safeguarding the Heart Child” have been conducted to tease out and delineate the processes of parenting children who have only recently been surviving their lethal heart condition [25]. Rempel and Harrison (2007) described a process of “safeguarding survival” through a study of mothers and fathers of young children from an early surgical cohort who were among the first in western Canada to benefit from life-saving surgery (born between 1996 and 2002). Given the absence of established care guidelines, limited home or community services for children with HLHS, these parents independently devised strategies to vigilantly safeguard their child's survival [26]. Both the mothers and fathers performed advanced nursing and medical assessments and procedures in their home. They made judgments regarding when to seek medical attention for their child while dealing with a demanding care regimen involving round-the-clock tube feeding, medication administration, and protecting their child from infection, especially during the first year of life. Parents also described strategies to safeguard their own well being, especially related to worry about their child's immediate and ongoing survival and associated parenting stress. All parents knew of other children with HLHS who had died and they described their awareness of their child's vulnerability to illness and further cardiac complications. Although all children were followed closely for developmental outcomes, parents surprisingly did not articulate concern about their children's actual or potential developmental impairment [27]. In fact, a key parenting strategy was finding a balance between optimistically appraising their child's progress and realistically perceiving their child as vulnerable [28]. These parents had been prepared for their child's possible death and were not worried about the progression of developmental milestones such as when their child started talking or walking [27].

Rempel recently studied parents and grandparents of children with HLHS from a later surgical cohort [29]. This cohort of children had a 2-year survival rate of 77%, compared to a 48% survival rate for the earlier cohort. This improvement is attributed to an alteration in the first surgical procedure and possibly by the introduction of a home monitoring program in which parents were given a weigh scale and an oximeter to daily monitor the baby's weight and oxygen levels [30]. In this recent cohort, parents focused less on the child's potential to die and were more aware of developmental impairments [31]; however, participants did not speak extensively about services received. Similar to Rempel's previous study, parents and grandparents felt that they were on their own to manage their child's HLHS once discharged from hospital, with no ongoing followup for changing needs beyond the time of diagnosis and initial treatment. Parents described their focus on immediate, day-to-day family functioning rather than a focus on pursuing services to address the long-term, developmental needs of their child or their own needs as parents. A key study outcome was the delineation of a process of “parenting under pressure” that provides a trajectory of parenting that complements the safeguarding strategies previously identified [29]. Parenting under Pressure was characterized by four overlapping and reemerging phases: (1) realizing and adjusting to the inconceivable, (2) growing increasingly attached, (3) watching for and accommodating the unexpected, and (4) encountering new challenges. A key recommendation of this study was to identify interventions for parents of children with complex health conditions move through the phases of Parenting under Pressure that may help them safeguard the survival of their children, as well as their own survival as parents as they manage multiple demands.

The purpose of this study was to conceptualize the needs of parents of young children with hypoplastic left heart syndrome (HLHS) to provide a theoretical framework to inform the development of future parent interventions. Specific research questions included (1) what are the common experiences and needs of parents of young children with HLHS? (2) How can these experiences and needs be synthesized to optimize translation of evidence for health care professionals? and (3) How can the resulting conceptualization inform future interventions?

## 2. Methods

### 2.1. Design and Participants

This research project, a secondary analysis of data from a grounded theory study [32], was conducted within a program of qualitative research that focused on developing theory of parenting children and adolescents with CHD to inform child, adolescent, parent, and family level interventions. A supra analysis is done using existing data when there are new but related research questions, and often new research methods are used [32]. Interpretive description was used as it is instrumental in developing an understanding of complex questions to inform clinical practice [33].

The larger grounded theory study from which Parenting under Pressure was conceptualized [29], involved parents and grandparents of young children with HLHS who had undergone a right ventricle to pulmonary artery surgical palliation shortly after birth. Parents were recruited through an advance practice nurse in a tertiary pediatric cardiac surgery program in western Canada. Parents recruited grandparents, who were contacted if they were interested in participating. Participants were 15 mothers, 10 fathers, 17 grandmothers, and 11 grandfathers of 15 children with HLHS. The children were 6 months to 4.5 years at the time of the original study and 10 were diagnosed antenatally. All children had the initial Sano surgery, 11 also had the Glenn surgery, and four had all three surgeries. Individual one- to two-hour interviews were conducted with the parents and grandparents and these were transcribed verbatim. Confidentiality of data among family members was maintained. Data was analyzed concurrently with data collection. Through the grounded theory analytic process, a hierarchical coding matrix was developed using constant comparative methods. Ethics approval was obtained through the university health research ethics board and covered the larger grounded theory study and this further analysis, as no further data were collected and the same data were analyzed within a similar time frame. In addition both interpretations delineated processes of parenting children with HLHS to further inform intervention.

### 2.2. Data Collection and Analysis

This project used four data sources for the interpretive description: (1) transcriptions, audio recordings, and field notes of the 53 previously conducted interviews, (2) conceptual memos written by the researchers as part of the analytic process, (3) knowledge from clinical practice in nursing young children with HLHS from the first author's experience, and (4) a review of descriptive and intervention studies in CHD.

**Figure 1:**
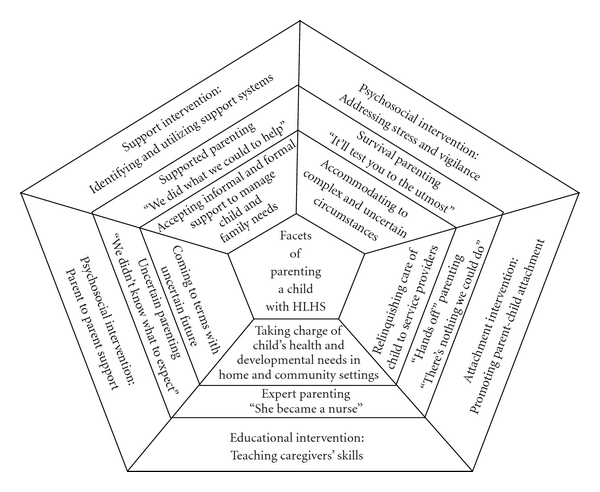
Theoretical model: Facets of parenting a child with hypoplastic left heart syndrome.

Research team members immersed themselves in the transcripts and digital recordings to identify themes in the parents' experiences and patterns in the data. The researchers wrote conceptual memos, had brainstorming meetings and repeatedly returned to the raw interview data and field notes to identify common themes. Codes of previously analyzed data that were relevant to the research question were read and reread. As common and disparate experiences of the parents were grouped, word searches were used to find words that closely identified the parenting experiences. A conceptual model was developed, which was then used as a framework for identification of interventions (see Figure 1). Finally, the framework was combined with the stages of the previously identified theory of Parenting under Pressure and related to clinical practice and current literature in pediatric cardiology.

We necessarily attended to aspects of rigour for our conduct of both the interpretive description and the secondary analysis. Foundational to a rigourous interpretative approach to research is an accounting of the researcher's role in data analysis and conceptualization. The first author's background in family nursing brought a resiliency lens to this research that was balanced by her advance practice nursing background with families of children with HLHS that sensitized her to the coexisting struggles and accomplishments of parents as they navigate the complex course of their child's illness and life-saving treatment. Additionally, the similar yet diverse clinical backgrounds of the second, fourth (pediatric occupational therapy and autism), and third authors (acute pediatric burns nursing in India) shaped our research interpretation in a holistic and comprehensive manner [34]. Rigour regarding the secondary analysis aspect of this study was achieved by having those involved with the original study involved with this study as well. Additionally, our research questions for this study were related to the research question and purpose of the original study.

## 3. Results and Discussion

### 3.1. Results

Persistent stress and uncertainty characterized parents' and grandparents' accounts. Parents understood the life-threatening nature of HLHS by knowing a child who died or by their own child nearly dying. Parents hoped for the best while simultaneously acknowledging the worst-case scenario. Despite their child's uncertain future, many parents felt lucky or grateful, especially in comparison to others, and described their personal growth through parenting a child with HLHS. Grandparents' “birds-eye” view of their adult child's experiences corroborated parents' accounts and provided additional insights.

#### 3.1.1. Facets of Parenting

A key goal of theory development is to identify variables that contribute to theoretical or explanatory models. Models generate hypotheses for further investigation and direct intervention [35]. Analysis of the interrelationships between codes that reflected the families' circumstances and their behaviours and interactions related to their child led to five facets of parenting a child with HLHS. The facets are cyclical and repeated, with some overlap as the child progresses through three surgeries. This conceptualization is not a categorization of families but of experiences of the families and has the potential to direct intervention.

#### 3.1.2. Survival Parenting: “It'll test you to the utmost”

Parents faced extraordinarily difficult conditions related to their child's precarious survival. Survival parenting involved accommodating to demanding, complex circumstances, and crises that were riddled with dilemmas and crossroads.

At the time of diagnosis, parents faced life-altering urgent decisions related to either a progressing pregnancy (antenatal diagnosis) or a critically ill baby (postnatal diagnosis). Parents had to consider all aspects of their life as they made a decision that would set the course for their baby's and family's life. As one father expressed: *“[I] wanted everything weighed out. [If we] go through with this*…*what kind of life is [child] going to have?…Are [our] lives going to be lived in hospital?”*

Survival parenting involved rearranging busy family and work lives to accommodate the new complex circumstances surrounding their sick baby. A mother expressed that many times she thought *“I cannot handle one more thing. [But] you do. You just do. You just kind of deal with it.”* A grandmother at the time of the first surgery said, *“I think it was a very, very scary time for them and stressful.”*

Survival parenting continued as their child's life was repeatedly at risk with further surgeries. One father expressed this by saying *“You do not realize how deep the well you are in until the next procedure comes along.”* Uncertainty about short- and long-term outcomes contributed to the stress and pressure. Although cautiously optimistic about the future, parents unquestioningly proceeded with life in the face of repeated surgeries, intensive monitoring and followup, and the constant demands of their family and work life. While parents were dealing emotionally with this stress, they often felt that they were powerless to help their baby while in hospital, which related to the facet of “hands-off” parenting.

#### 3.1.3. “Hands-Off” Parenting: “There's nothing we could do”

This facet related to the staged surgeries for HLHS and the professional's role that often excluded parents. Professionals relieved parents of many aspects of caring for their baby with positive intentions to save the baby and “rescue” the parents by disburdening them. Even as early as the antenatal diagnosis, one father expressed his hurt at being left out at his wife's 24-week diagnostic ultrasound when *“they have people running in and out of this room that looked all panicky, and there's nothing I can do about it… I have to sit and wait.…I was the second class citizen. I want to know what's going on*…*and they won't say anything to me.”* With an antenatal diagnosis, “hands-off” parenting began immediately after the birth when the baby was immediately removed from the delivery room by health care professionals while the mother and father were encouraged to *“make it over [to the tertiary centre] when you can.”*

While parents acknowledged the vital role of health care professionals in their child's life, “hands-off” parenting was not what these parents had anticipated.

*You expect to have a child and take them home and do all these wonderful things for them, so when she was hospitalized, we didn't get to do anything for her.* (Mother)

Parents were often encouraged not to touch the baby, as shown by a father who said, *“if I had my hand on her head and I moved my finger 1/2 inch, the nurse would say [to stop]*…*as it might irritate him.”* Another father said: *“The first four months was all hospital and I don't really count that as us parenting.”* In contrast, a third father viewed his role as important even within the restrictions. *“You watch her facial expressions and you learn to read her eyes and her body movement and you hold her hand a lot… you can sing to her, talk to her. … You can do parenting stuff."*

The actions that tended to remove the parents from a meaningful role with their baby seemed related to the individual nurse's values and beliefs about what was best.

*We got one nurse who thought she knew everything-baby sleeps best in the crib. Best not being held, and so on. Really regimented*…*and then we had* …*the one that trains the nurses in NICU, basically gave us the “I've taken the vitals, I've given her meds, she's yours.” And I'm like “Excuse me.” Cause the day before I've been fighting with the nurse just to hold her!* (Father)

Even after parents had gained hands-on experience and confidence in the care of their child in their home setting and felt they understood their child better than the health care professionals, they were extricated from their parenting role when their child returned to the hospital for subsequent surgeries.

*I felt they were pumping him full of way to much stuff to calm him down. They wouldn't let us pick him up because he's intubated, and I felt that was all he really needed.* (Mother)

Grandparents also expressed concern for the hospitalized child related to “hands-off” parenting.

*There's no place for the parents to sleep*…*we have to think of the anxiety that these children have, coming out of an operation and not having mommy there,*…*the anxiety of leaving your child and that child not seeing you*…*but if they can see mommy there*…*I think you're going to get children that are going to heal much better and you're not going to have the anxiety of either parent or child. *

#### 3.1.4. Expert Parenting: “She became a nurse”

The parents, and to a lesser yet significant extent the grandparents, became knowledgeable about HLHS and proficient regarding care. The term expert means practiced, experienced, qualified, skilled, savvy, and professional [36]. Becoming an expert usually implies acquiring knowledge/skill over time. In contrast, the diagnosis of life-threatening CHD moved ordinary expectant parents into an exigent circumstance that demanded immediate expertise. Parents quickly took charge, gained knowledge and understanding for decision making, and practical hands-on skills so that they could take their baby home. One grandmother reported *“[Mom] probably knows as much as a junior nurse does.”* A parent with a health care background described her experience.

*I don't feel like I've been on maternity leave. I feel like I have never left my job*…*I am able to do more as a mom than I was as a [health care professional]. *

Expert parenting included being knowledgeable about HLHS: prognosis, treatment options, including which centers had the best survival rates, as well as what was normal or not for their child and how to respond. The time and support for learning skills in the hospital were limited. Once home parents were abruptly on their own. One parent described the medications: *“27 syringes of meds in a day. It took us about an hour each night to sit down, and draw up all the meds…ready for the next day.”* Parents became experts regarding nasogastric tubes and enteral feeds and were vigilant about medications, fluid monitoring, daily weights, heart auscultation, and oximetry readings.

A striking finding was that, aside from their own parents, the grandparents in this study, affirming their expert parenting, the parents did not describe others recognizing or affirming their expertise. Parents expressed concern and pride that they knew more about complex CHD than the generalist health professionals in their home communities. They became skilled in navigating the system and advocating for their child. While this vigilance was often necessary, it was difficult for the parents to decrease their vigilance as their child improved and perhaps needed less close monitoring. There were dilemmas as they tried to balance appropriate vigilance and worry.

*You're solely responsible*…*we were supposed to know when her function was bad or not. That responsibility is huge*…*. That was one of our biggest challenges*…*was being responsible and knowing but not being, not being overly worried either*…*kind of have to keep a nice balance.* (Mother)

Often the parents' expertise was not recognized or valued when they sought medical attention for their children in their home communities. Parents described attempts to obtain medical attention for their child who they knew needed help, only to have their concerns dismissed as not urgent. *“The doctor said he did not need to see her…. She finally turned blue and 911 was called…. It would be nice if the doctors would realize that [the parents] are with her 24 hours a day, and they know when something is off.”* This constant parental worry and vigilance were often warranted, as the parents were continually faced with the uncertainly that accompanied their child's precarious survival.

#### 3.1.5. Uncertain Parenting: “We didn't know what to expect”

Right from the time of the child's diagnosis, parents expressed that *“you don't really know what's in store for you.”* This facet involved living with repeated times of increased uncertainty. Even though parents became experts in understanding their child and monitoring the child's condition, they were continually having to face uncertainty as the child returned for further surgeries or had complications or setbacks. Uncertainty continued as the child underwent a second surgery at 4–6 months of age and then a third surgery at 3–5 years of age. Faced with knowledge of surgical mortality, parents who were awaiting their child's surgery were anxious as *“he's going to have another surgery, and this is the tough one, I hear.”* One father awaiting the third surgery reported.

*It's going to be all those feelings that we had before brought on tenfold*…*there's a chance that things might go wrong*…*. it's been hard for [wife] to make plans for the future because she's worried that [daughter with HLHS] might not be there.*…*I always think that things are going to work out*…*. We're going to be there for two weeks then we're going to be back home, back to normal again like nothing ever happened. *

Another father expressed what it was like to face this uncertainty.

*We basically thought he was gone. I remember thinking*… *it's amazing he's made it this far. And it was almost calming [to think] he was going to go…But then he made it again. No one was happier [than me] that he pulled through totally. *

Uncertain parenting, while demonstrating parental resilience, also illustrated parental stress and vulnerability: a mixture of hope and worry especially as parents anticipated what life would be like for their child in the future.

*You try to stay positive but the reality of it is I think that you have to deal, in your head…with the good and the bad.* (Mother)

Even after the child has survived the surgery, a father described the worst thing is that *“every night you put him down, you hope the next morning he's going to be yelling ‘Dad' or ‘Mom' out of the crib.”* Grandparents also faced this uncertainty, and their concern for their grown child and grandchildren contributed to the final facet, extended parenting.

#### 3.1.6. Supported Parenting: “We did what we could do to help”

Supported parenting is best contextualized in relation to the pressures and demands that the parents encountered. They were at risk for not being able to accommodate to complex circumstances (survival parenting), relinquish their parenting role (“hands-off” parenting), become an expert (expert parenting), and live with constant uncertainty (uncertain parenting) without the emotional and instrumental support of others. Grandparents were often the ones to whom parents turned first. Grandparents were more intensely parenting their adult children than would be expected in terms of time, magnitude, and flexibility even though they were often working and living in neighbouring provinces.

Parents sought and accepted help from extended family, especially grandparents, who listened to the parents, cared for siblings, and provided short-term respite for the child with HLHS when possible. They would often trade off with the fathers between managing the “home front” including siblings or supporting the mothers when fathers had to return to home to work and to attend to their home and the bills. Grandparents were especially concerned about how a sick child would affect their adult child's marital relationship and some of their assistance was to safeguard that couple relationship.

The extraordinary nature of the parenting was further described by the grandparents who provided additional information about the parents such as a parent's previous or present mental health condition and traumatic experiences including the death of a parent or friend. Grandparents repeatedly articulated how impressed they were with how well their children managed family life with a child with HLHS and acknowledged that the demands could exceed the parents' resources. Parents retained their parenting role, as grandparents deferred to them and their partners for decisions around the baby and their role. The grandparents described their boundaries: *“I know he's not my, mine but it's really hard for me to not give advice”* and *“I'm not an outsider, but not an insider. I'm just a grandparent and I enjoy that part cause I love spoiling the kids”* and with regards to discipline, *“That's not my job. I did that with mine. I don't have to do it with theirs.”*

A wonderful moment for grandparents was when their adult children began managing all aspects of the child's care and life. A grandmother recounted her joy when she observed this even though it meant that she had to step back.

*They started to parent. They started to tell me what to do. At first I was insulted because I thought “Wow! Come on you guys! I was there the whole time! I've been here since you found out!”*…*and then I thought “What are you doing?! That's their baby.” *

### 3.2. Discussion

This interpretive description delineates five facets of parenting. Much of the research on parents of children with CHD has focused on parental stress [37, 38] and mental health, [23, 39, 40] the influence on child outcomes of parenting style [17, 41, 42], and parental overprotection [43]. Our findings purport an alternative view of the role of parents and parenting in the lives of their child with CHD. This view affirms parental resilience and directs health care practitioners to offer evidence-based interventions to support parents within the context of the family. As high technology care for children with complex CHD continues to be offered and even expanded despite our increasingly constrained health care environment, evidence of intervention effectiveness and feasibility for parents who are caring for their children with minimal supports is needed [44, 45]. Based on Sidani and Braden's model of intervention design [46] therefore, the empirically based facets of parenting become the *critical components for intervention* to address facet-specific issues. The facet model itself is a dynamic representation, open to further expansion and development for this and other populations. Additional facets can be added to make the shape a hexagon (6 facets), for example.

Our current model depicts parenting facets in the shape of a pentagon gem (see Figure 1). The inner ring houses the key facet themes. These were labelled in the next outer ring, along with a meaningful and succinct parent quote to describe the facet. The outer ring depicts the key components of parental interventions that are yet to be determined.

Given the phased trajectory for children with complex CHD, including HLHS, as previously delineated by the multiphased process of parenting under pressure (PUP), the facets of parenting are best considered in relation to the PUP phases. The facets of parenting when mapped onto the four phases of Parenting under Pressure have provided direction to articulate guiding principles for developing parent interventions (Table 1). The facet-specific interventions during the initial phase of *adjusting to the inconceivable* are the most directive, possibly emulating crisis intervention. Survival parenting can be supported by validating emotional parent responses related to the diagnosis of the child's CHD [47]. Expert parenting can be supported by providing information about treatment options to facilitate parental decision making following diagnosis [48, 49]. Concerning “Hands-off” parenting an advocacy intervention by nurses to ensure that the parents have even a brief time to meet and hold their baby in the delivery room before the baby is transferred to specialized care is warranted. Identifying and mobilizing support for parents in the early stages of their adjustment to the devastating diagnosis of CHD addresses the facet of supported parenting and determining the appropriate timing and content of an education/information intervention, for example, have the potential to address uncertain parenting [50, 51]. A parent support intervention to address the challenges of survival and uncertain parenting is needed to assist parents to monitor and address their own mental health and develop a network of formal and informal supports. Improved mental health outcomes for mothers of infants with CHD following educational-behavioural intervention during their neonate's hospitalization [52] provides direction for intervention that could be extended to fathers as well and beyond the neonatal period.

Guiding principles for developing multifaceted interventions for parents during the phase of *growing increasingly attached* include less direct approaches such as facilitating development of appropriate vigilance (survival parenting), knowledge about the baby's condition (expert parenting) and coaching parents to advocate for themselves to be as “hands-on” as possible in the hospital setting. Research regarding understanding of CHD in parents of children of all ages (median age 6 years) identified knowledge gaps concerning the heart defect and medications [53]. After the child survives early surgical intervention, parents need to learn about the effects of cardiac malformations on children both physically and emotionally/psychologically and how to support the child's development [28, 54].

As parents prepare to care for their child at home after each surgery, interventions oriented towards empowering parents to responsively *accommodate to the unexpected* are necessary to address parents' need to provide situation-appropriate vigilance (Survival parenting), confidently use monitoring equipment in their home settings (Expert parenting), assertively draw forth support, both formal and informal (Supported parenting), and including parent-to-parent support (Uncertain parenting). Interventions during the phase of *encountering new challenges* necessarily relate to ensuring that parents can sustain their caregiving and parenting responsibilities and challenges over time. As parents anticipate the child's entry into the school system knowledge about the heart condition and how to convey this information to teachers is vital [54, 55].

Encouraging respite (Survival parenting), providing relevant, comprehensive and evidence-based information about their child's condition and required care and monitoring (Expert parenting), supporting parents in navigating the system (Supported parenting) and ensuring that interventions are offered to parents at all stages of their child's illness trajectory (Uncertain parenting) will address the range of parent need as specified by the facets of parenting.

The facet of supported parenting directs us to consider a social support intervention that includes constructing a genogram (i.e., a visual depiction of family structure) and ecomap (i.e., a visual depiction of sources of support and nonsupport) [47] at the time of diagnosis to determine the family's life lines and encourage immediate support mobilization. At follow-up appointments, the family's genogram and ecomap can be reviewed to affirm actual support and jointly identify potential sources of further social support. Intervention focused on parenting expectations are recommended for parents of children diagnosed with chronic medical problems to assist the parents in the process of adaptation [56].

## 4. Conclusion

Development of a model depicting the *facets of parenting* that translates into the critical components for intervention is a vital step towards innovative and effective practice development in pediatric cardiology. The integration of the facets of parenting with the phases of Parenting under Pressure in the form of guiding principles for developing interventions provides a framework to identify interventions that are in existence in clinical settings and in the published literature and the intervention gaps. Future research involves the identification and pilot testing of tools to measure intervention outcomes such as parent knowledge, parent resilience, parenting stress, and family functioning. In developing interventions for parents in stressful circumstances, it is critical to engage stakeholders in the development of timely and appropriate interventions and target interventions to the engaged groups [57]. Thus a further step towards intervention includes eliciting data from parents and health care professionals regarding the timing of intervention, the method of delivery (e.g., clinic-based, web-based, peer-support, telephone, videoconference) and the guiding principles for intervention development that have incorporated the facets of parenting and the theory of parenting under pressure. Applying this integrated evidence in combination with a review and synthesis of the published intervention research in pediatric cardiology will contribute to evidence-informed practices to support parents so that they are well situated to optimize outcomes for their child with complex CHD.

## Figures and Tables

**Table 1:** Guiding principles for developing multifaceted interventions.

→	Phases of Parenting under Pressure			
↓Facets	Adjusting to the inconceivable	Growing increasingly attached	Accommodating to the unexpected	Encountering new challenges
Survival parenting	Validate emotional responses	Facilitate development of appropriate vigilance	Coach parent to provide situation-appropriate vigilance	Encourage respite
Expert parenting	Offer information to facilitate parental decision-making following diagnosis	Facilitate increasing knowledge about baby's condition while in the hospital	Facilitate process of parent becoming the expert including their use of a weigh scale and oximeter	Translate relevant and comprehensive knowledge regarding care of child with CHD to parents over time
“Hands-Off” parenting	Advocate for parents to have even a brief time with their newborn in the delivery room before transfer to pediatric centre	Coach parents to advocate for themselves to be as “hands-on” as possible even critical care settings	Respect and utilize parental expert knowledge and role when child returns to hospital for subsequent procedures and surgeries	Value parent involvement
Supported parenting	Identify and mobilize support	Encourage parents to involve extended family during hospitalization	Facilitate parent process of drawing forth support, both formal and informal	Support parents in navigating the system
Uncertain parenting	Determine appropriate timing of intervention	Facilitate expression of grief responses through the telling of illness narratives (i.e., events around time of diagnosis)	Encourage parent-to-parent support	Supporting parents overtime
